# Bridging borders: Current trends and future directions in comparative health systems research

**DOI:** 10.1111/1475-6773.14385

**Published:** 2024-09-25

**Authors:** Nicholas Bowden, Jose F. Figueroa, Irene Papanicolas

**Affiliations:** ^1^ Department of Women's and Children's Health University of Otago Dunedin New Zealand; ^2^ Department of Health Policy and Management Harvard T.H. Chan School of Public Health Boston Massachusetts USA; ^3^ Department of Health Services, Policy and Practice Brown University School of Public Health Providence Rhode Island USA

## 1 INTRODUCTION

Over the last two decades, comparative health systems research has gained significant traction as policymakers and researchers seek to better understand how to improve the effectiveness and efficiency of health‐care systems worldwide.^1^ While most studies undertaken to achieve these goals continue to be predominantly at the national or sub‐national levels, the role and importance of cross‐country comparison research is increasingly being acknowledged. Recent challenges such as the COVID‐19 pandemic, inflationary pressures, rising health‐care costs globally, climate change, and decreasing life expectancy among several high‐income countries^2^, ^3^ have increased the importance and urgency of this work. Collaborative research efforts across disciplines and countries are therefore needed to identify focused solutions that health systems can apply to the challenges they currently face, and those that may arise in the future.

A range of entities have risen to meet this challenge by producing harmonized metrics and analyses from which to begin to answer these questions. These range from intergovernmental organizations such as the World Health Organization (WHO), the Organization for Economic Co‐operation and Development (OECD), the World Bank, and the European Observatory on Health Systems and Policies to foundations including the Commonwealth Fund and the Health Foundation. However, academic organizations also have an important role to play in closing gaps in data collection, advancing methods and collaboration across disciplines and countries, and producing robust analyses to inform key policy questions.

In this editorial, we summarize the current state of cross‐country comparison work at a high level, outline research gaps that remain, and discuss the contribution to this literature of research contained in this special section on international comparisons published in *Health Services Research*.

## 2 CURRENT STATE OF CROSS‐COUNTRY COMPARISON WORK

Efforts that contribute to comparative health systems research can be broadly grouped into measurement of health‐related information — spearheaded by intergovernmental organizations — and health comparisons which are increasingly being led by key foundations and university‐based institutes.

The current state of health measurement can be further categorized into three key areas: health outcomes, health inputs and expenditures, and health systems. Evidence‐based insights that guide global health change require accurate measurement in each of these areas. The WHO plays a crucial role in outcome measurement through its Global Health Observatory, which includes the annual *World Health Statistics* report, compiling data on key health indicators, helping to monitor global health trends.^4^ The Institute for Health Metrics and Evaluation (IHME) also significantly contributes by providing detailed metrics on disease burden, mortality, and risk factors, informing public health policies and resource allocation.^5^

Measurement of health inputs and expenditures, essential for understanding the efficiency and sustainability of health systems, is driven by the World Bank, the WHO, and the OECD. For example, the WHO's *Global Health Expenditure Database* (GHED) offers comprehensive data on health expenditure by source and function, crucial for tracking financial flows in health systems.^6^ Additionally, the *System of Health Accounts* (SHA), developed collaboratively by the WHO, the OECD, and the EU, provides an internationally standardized framework for compiling health expenditure data, ensuring consistency and comparability across countries.^7^ The OECD's *Health Expenditure and Financing Database* further supports this by offering detailed data on health spending across its member countries.^8^

Finally, health systems measurement is vital for identifying best practices and informing policy decisions. The OECD plays a pivotal role in this through its *Health at a Glance* series and the OECD Data Explorer, which offer comprehensive data on health‐care quality, access, and expenditure across countries.^9^ The WHO's *Health System Performance Assessment* frameworks help countries evaluate the efficiency, equity, and effectiveness of their health systems.^10^ The European Observatory on Health Systems and Policies' *Health Systems in Transition* series provide in‐depth analyses of health systems in Europe and other OECD countries, covering aspects like organization, financing, and service delivery, offering essential insights for policymakers aiming to improve health‐care systems.^11^, ^12^

Increasingly, key foundations and university‐based entities contribute to this body of work through their specialization in cross‐country comparison studies to help inform local narratives and policy change. For example, the Commonwealth Fund's international health policy surveys assess and compare health system performance across 10 countries,^13^, ^14^ and reports such as *Mirror*, *Mirror on the Wall* offer detailed insights into comparative performance of the United States health system to other high‐income countries.^15^ The Health Foundation also plays a significant role through its examination of how the National Health Service compares with health systems in other countries, exploring differences in health‐care spending, life expectancy, and health outcomes across countries.^16^, ^17^

University‐based research centers and collaboratives are also increasingly making contributions to comparative health systems research. The Observational Health Data Sciences and Informatics is an initiative that enhances international health comparison by harmonizing global observational health data and enabling large‐scale, cross‐country research.^18^ Similarly, the European Collaboration of Healthcare Optimization, the Population Health Information Research Infrastructure, and Innovative, Non‐invasive and Fully Acceptable Exploration Technologies are Europe‐focused initiatives that collectively improve consistency and comparability of data and undertake a range of collaborative cross‐country research.^19^, ^20^, ^21^, ^22^ More recently, international collaboratives that compare the United States with other high‐income countries, including the International Health System Research Collaborative and the International Collaborative on Costs, Outcomes, and Needs in Care, have also emerged with their research focusing on evaluating differences in care trajectories of comparable high‐need, high‐cost patient subpopulations.^23^, ^24^, ^25^, ^26^, ^27^, ^28^, ^29^

## 3 RESEARCH GAPS

While significant advances in comparative health systems research have been made, a number of challenges and research gaps remain.^30^ First is the ongoing challenge for standardized data collection methods and metrics. Despite significant advances, the lack of uniformity in data collection and measurement can still hinder the comparability of findings across countries and potentially exaggerate or obscure differences in health system performance that are more reflective of variations in measurement practices than actual performance disparities.^1^, ^23^, ^30^

In addition, health systems remain poorly understood, with existing data providing only partial insights into their functioning and organization.^31^ Most analyses focus on specific aspects like clinical care for subgroups of patients or technology cost‐effectiveness, usually within a single country, making it difficult to gain a comprehensive understanding of entire systems. Moreover, the organization of care delivery varies significantly across countries and is influenced by both national and international organizations. To better analyze and compare health systems globally, we need a better framework and understanding of key differences in the organization of health systems across countries that can serve as a foundation for comparative health systems work. Significant progress is being made collecting necessary health system data including the new round of the OECD Health System Characteristics survey of 33 countries, and the European Observatory on Health Systems and Policies' Health Systems and Policy Monitor. As more data becomes available on health system structures, this framework could be used to help to describe and measure how these functions are implemented across different countries and to examine their relationship with health system performance.

There is also a pressing need for more longitudinal studies that explore variation of health systems and performance over time.^1^ This has never been more important than in the context of the COVID‐19 pandemic which revealed significant variations in health system preparedness, response strategies, and resulting health outcomes.^32^, ^33^ A nuanced understanding of temporal variation in health system performance within and across countries is vital for enhancing future health system resilience and effectiveness. In particular, an increased focus on identifying whether certain types of health systems consistently outperform others in terms of resilience, sustainability, and overall performance is required. Understanding these patterns could provide valuable insights into designing more robust health systems that are better equipped to handle future challenges.

Lastly, more work should be undertaken to incorporate the social determinants of health into comparative analyses. Health systems do not operate in isolation; they are influenced by a range of social, economic, and environmental factors.^34^ Integrating these determinants into health systems research provides a more holistic understanding of health outcomes and can inform more effective policy interventions. This is particularly relevant in the context of persistent and worsening global health inequities and the important role that social determinants of health can play in reducing these inequities.

## 4 CONTRIBUTION OF THIS SPECIAL SECTION ON INTERNATIONAL COMPARISONS

The papers in this special section each make valuable contributions to these research gaps. Papanicolas et al.^35^ offer important insights into a critical aspect of cross‐country health comparison measurement by introducing a novel approach to deflating health expenditure and examining the dynamics of health price growth across the United States, Australia, Canada, France, and the Netherlands from 2000 to 2020. This research demonstrates the profound impact that different price indices can have on health expenditure analyses. The study reveals that the United States experienced the highest cumulative health price growth relative to general price growth. It also highlights that price growth for health services funded by public payers was generally higher than for those funded by households. The findings suggest that general price indices likely underestimate the contribution of health price growth to overall health expenditure increases and underlines the necessity for more accurate health‐specific price indices to inform policy and better manage health expenditures across countries.

The paper by Kyriopoulos et al.^36^ provides important insights in an important social determinant of health by investigating wealth‐related disparities in self‐reported health among older adults across 15 high‐income countries. This study enhances our understanding of health determinants in cross‐country comparisons, revealing substantial wealth‐related health inequalities. Notably, the United States exhibits the highest levels of inequality compared with the European countries included in the study. These disparities persist over time and across different age groups. While the authors rightly conclude that the study underscores the importance of addressing socioeconomic disparities to enhance overall health outcomes and reduce health inequalities, it also serves as a strong example of the need to integrate social determinants of health into cross‐country analyses.

The paper by Ledesma et al.^37^ fills a crucial research gap by comparing how different global health systems managed disruptions in hospitalizations and ambulatory care during the COVID‐19 pandemic. While prior research has often focused on single‐country analyses or specific conditions, this study takes a broader approach, using time‐series data from 26 countries to assess the scale and variability of disruptions across diverse health systems. The study finds that the pandemic was associated with significantly reduced non‐COVID‐19 hospitalizations, avoidable hospitalizations, and surgical procedures, with considerable cross‐country variability. Factors such as workforce per capita, insurance coverage, and hospital beds were linked to fewer disruptions, whereas stricter COVID‐19 measures and higher excess mortality were associated with greater disruptions. In the context of the social determinants of health a notable finding from the study is that higher‐income inequality was linked to greater disruptions in surgical care. This study underscores the importance of health system preparedness and adaptability, offering critical insights for future policy planning to enhance resilience against global health crises.

Finally, Bowden et al.^38^ present an international comparison of hospitalizations and emergency department (ED) visits related to mental health conditions across eight high‐income countries before and during the COVID‐19 pandemic. To date, there is limited evidence evaluating how high‐income countries differ in the management of these populations when they present for care in acute care settings. The study highlights significant cross‐country variations in acute mental health‐care utilization, with the United States displaying the highest combined rates of hospitalizations and ED visits, while Finland exhibited the lowest. Importantly, the research identifies shifts in care settings during the pandemic, particularly in the US, where there was a notable increase in inpatient care and a corresponding decrease in ED visits. The study's utilization of time series data before and during the COVID‐19 period, in addition to the examination of multiple care settings, serves an important contribution to exploring how system organization influences site of care.

## 5 THE WAY FORWARD

The studies presented in this special section offer important contributions to the understanding of how different health systems respond to global challenges and disparities. They collectively demonstrate the power of comprehensive, cross‐country analysis in uncovering significant variations in health outcomes and system resilience. These findings, derived from diverse data sources and methodologies, underscore the value of international collaboration and comparative research in health policy. The insights gained not only highlight areas of strengths and weaknesses within specific countries but also provide a roadmap for enhancing global health system preparedness and equity.

As we look ahead, leveraging these comparative approaches will be an important component of the research evidence to inform the design of policies that can more effectively address the complexities of global health. However, while opportunities to identify potential improvements in health systems exist, such research must be accompanied by a deeper understanding of the reasons behind differences. Data limitations must be considered in both the analysis and interpretation to ensure accuracy and relevance. One option that could be further explored is the use of Federated Data^39^ and synthetic data generation^40^ as key methodological approaches that may mitigate some of the key limitations that have been noted in this work. These approaches allow for data sharing and analysis across different jurisdictions that ensure measurement and methodological consistency while still preserving privacy and security of data. Finally, results from comparative research should be interpreted within the context of nuanced understandings of national policies, values, and priorities to ensure that they are meaningful and actionable.

## Data Availability

There are no data to be made available.
